# USP26-mediated stabilization of RAD51 promotes endometrial cancer progression and limits ferroptosis

**DOI:** 10.3389/fonc.2026.1896160

**Published:** 2026-09-18

**Authors:** Jiayu Jing, Qi Xie, Shuang Jing, Na Li, Yangyang Wang, Yunfeng Zhang, Yue Wang

**Affiliations:** 1 Department of Gynecology, Henan Provincial People’s Hospital, People’s Hospital of Zhengzhou University, Zhengzhou, China; 2 Department of Pathology, Henan Provincial People’s Hospital, People’s Hospital of Zhengzhou University, Zhengzhou, China

**Keywords:** deubiquitination, endometrial cancer, ferroptosis, RAD51, tumor progression, ubiquitination, USP26

## Abstract

**Background:**

Ferroptosis is increasingly implicated in endometrial cancer, but the role of the homologous recombination protein RAD51 in ferroptotic stress and its post-translational regulation remain unclear.

**Methods:**

RAD51 expression was assessed in GEO datasets, UCEC tissues, and endometrial cancer cell lines. RAD51 loss-of-function was evaluated using proliferation, cell-death, Transwell, ferroptosis-related, and xenograft assays. IP–MS, co-immunoprecipitation, cycloheximide chase, proteasome inhibition, ubiquitination assays, USP26-C304S, and RAD51 rescue experiments were used to define USP26-dependent RAD51 regulation.

**Results:**

RAD51 was upregulated in endometrial cancer and its depletion reduced proliferation and xenograft growth, lowered Transwell migration/invasion readouts, and increased ferroptosis-associated changes. USP26 associated with RAD51, reduced its ubiquitination, and stabilized RAD51 in a catalytic activity-dependent manner. RAD51 re-expression partially rescued the effects of USP26 depletion.

**Conclusions:**

The USP26–RAD51 axis supports endometrial cancer progression and limits ferroptosis-associated stress, identifying RAD51 protein stability as a potential mechanistic vulnerability.

## 1 Introduction

Endometrial cancer is a common gynecologic malignancy. Although surgery is effective for most early-stage disease, outcomes remain poor for advanced, recurrent, or aggressive tumors, reflecting substantial molecular and biological heterogeneity (1–4). Identifying tumor-cell survival mechanisms may therefore reveal new therapeutic vulnerabilities.

As an iron-dependent form of regulated cell death, ferroptosis arises from oxidative lipid damage and insufficient protection by antioxidants (5, 6). The SLC7A11–GSH–GPX4 axis suppresses ferroptosis, whereas ACSL4 promotes ferroptosis sensitivity; p53 can also modulate this process through cystine metabolism and SLC7A11 (7–10). Altered ferroptosis sensitivity has been linked to tumor progression and therapeutic resistance, including in endometrial cancer (11–17), but its molecular determinants remain incompletely defined.

RAD51 is a core homologous recombination recombinase that maintains genomic integrity and supports tumor-cell survival under replication stress and DNA damage. DNA repair proteins can also participate in broader stress-response programs (18, 19). Whether RAD51 influences ferroptosis susceptibility in endometrial cancer, and how its protein abundance is maintained, remain unclear.

RAD51 is regulated by post-translational modifications. UCHL3 deubiquitinates RAD51 to facilitate BRCA2 interaction, whereas FBH1 and RFWD3 ubiquitinate RAD51 to regulate its localization and removal from DNA-damage sites (20–23). USP26 is a deubiquitinase with context-dependent functions in several physiological and cancer settings (24–29), but its role in endometrial cancer and its relationship with RAD51 have not been defined.

We investigated the role of RAD51 in endometrial cancer ferroptosis and the mechanisms controlling its protein stability. RAD51 immunoprecipitation followed by LC–MS/MS was used to identify candidate deubiquitinases, followed by biochemical, catalytic-mutant, and rescue analyses of USP26. This design tested whether USP26-dependent RAD51 stabilization contributes to ferroptosis resistance and tumor progression.

## 2 Materials and methods

### 2.1 Bioinformatics analysis

Public transcriptomic datasets were used to assess RAD51 expression and associated pathways in UCEC. RAD51 expression in tumor and non-tumor endometrium was compared in GSE17025 and GSE146889.

For GSVA, GSE106191 and GSE17025 samples were divided into RAD51-high and RAD51-low groups using the cohort median, and pathway activity was compared between groups.

For independent Hallmark GSEA validation, genes in GSE106191, GSE119041, GSE146889, and GSE17025 were ranked by their Pearson correlation with RAD51 expression and analyzed against MSigDB Hallmark gene sets using clusterProfiler. Detailed parameters and significance criteria are provided in the **Supplementary Methodology**.

RAD51 correlations with representative ferroptosis-related genes were assessed in TCGA-UCEC, and USP26–RAD51 mRNA correlations were evaluated in GSE106191 and GSE17025.

**Supplementary materials** provide the methodology, key parameters, and result files for the bioinformatics analyses. The source GEO and TCGA datasets are publicly available as specified in the manuscript.

### 2.2 Clinical specimens and immunohistochemistry

Forty primary UCEC tissues and 12 adjacent non-tumor endometrial tissues were collected from patients undergoing surgery at Henan Provincial People’s Hospital. Patients with histologically confirmed primary UCEC and adequate tissue and clinicopathological data were included; those receiving preoperative antitumor therapy or with insufficient tissue were excluded. We examined formalin-fixed, paraffin-embedded (FFPE) tissue specimens. The study was granted approval by the institutional Ethics Committee (No. 2021-80), and every participant supplied written informed consent. Clinicopathological features are summarized in **Supplementary Table S1**.

### 2.3 Immunohistochemistry

Paraffin sections were deparaffinized, rehydrated, subjected to antigen retrieval, and incubated overnight at 4 °C with antibodies against RAD51, USP26, Ki-67, ACSL4, GPX4, or 4-HNE. Staining was visualized with DAB and hematoxylin. Two pathologists blinded to clinicopathological data independently scored staining intensity (0–3) and positive-cell proportion (0–4); the final IHC score was their product, with discrepancies resolved by consensus. Tumor versus adjacent-tissue scores were compared by Mann–Whitney U test, and USP26–RAD51 IHC scores by Spearman rank correlation. Antibodies are listed in **Supplementary Table S2**.

### 2.4 RNA interference, plasmid transfection, and generation of stable cell lines

Transient RAD51 or USP26 knockdown used two independent siRNAs per gene with non-targeting siRNA controls. Gain-of-function experiments used RAD51, USP26-WT, USP26-C304S, or empty-vector plasmids. Stable RAD51-knockdown HEC-1A cells were generated with sh-NC, sh-RAD51#1, or sh-RAD51#2 lentiviruses followed by antibiotic selection. All vectors and oligonucleotides were obtained from Shanghai GeneBio Co., Ltd. The USP26-C304S mutant (NM_031907.3) contained a Cys304Ser substitution. Knockdown/overexpression efficiency was verified by qRT-PCR and western blotting; sequences are provided in **Supplementary Table S3**.

### 2.5 Quantitative real-time PCR

Total RNA was isolated, reverse-transcribed into cDNA, and subjected to qRT-PCR with a fluorescent master mix. Target gene expression was normalized to an endogenous reference, and relative quantities were determined using the 2^−ΔΔCt method. Primer sequences are listed in **Supplementary Table S3**.

### 2.6 Western blotting

Proteins were harvested using a lysis buffer supplemented with protease and phosphatase inhibitors, followed by concentration determination, resolution via SDS-PAGE, and electroblotting onto membranes. Subsequent to blocking, the membranes underwent incubation with specified primary and secondary antibodies, with detection achieved through chemiluminescence. Signal intensities were measured via image analysis and normalized against β-actin levels. A comprehensive list of the antibodies employed is provided in **Supplementary Table S2**.

### 2.7 CCK-8 assay

After transfection, KLE and HEC-1A cells were plated into 96-well formats at a density of 3 × 10³ cells per well. Following a 2-hour incubation with the CCK-8 solution, optical density was recorded at 450 nm at intervals of 0, 24, 48, 72, and 96 hours. Data derived from five technical replicates per experimental condition were averaged across three biologically independent trials.

### 2.8 EdU incorporation assay

After transfection, cells were seeded on coverslips at 5 × 10^4^ cells/well and cultured for 48 h, then labeled with 10 μM EdU for 2 h, fixed, permeabilized, fluorescently labeled, and counterstained with DAPI. Five random fields per sample were analyzed, and EdU-positive cells were expressed as a percentage of DAPI-positive nuclei. Three independent experiments were performed.

### 2.9 TUNEL assay

After transfection, cells were seeded on coverslips at 5 × 10^4^ cells/well and cultured for 48 h, then fixed, permeabilized, stained with a TUNEL kit, and counterstained with DAPI. Five random fields per sample were analyzed, and TUNEL-positive cells were expressed as a percentage of DAPI-positive nuclei. Three independent experiments were performed.

### 2.10 Transwell migration and invasion assays

Cellular migratory and invasive capacities were evaluated using 24-well Transwell assemblies equipped with 8-μm pore membranes. To assess migration, $5 \times 10^4$ cells suspended in 200 μL of serum-free medium were seeded into the upper compartments; conversely, invasion assays utilized inserts pre-coated with Matrigel, into which $8 \times 10^4$ cells were introduced. The lower chambers were filled with 600 μL of culture medium supplemented with 10% fetal bovine serum (FBS) to serve as a chemoattractant. Following incubation periods of 24 hours for migration and 24–36 hours for invasion, cells adhering to the underside of the membranes were fixed, stained, and enumerated across five randomly selected microscopic fields. Data represent the mean values derived from three technical replicates per condition, aggregated from three biologically independent experiments.

### 2.11 Detection of intracellular and mitochondrial ferrous iron

Intracellular and mitochondrial Fe^2+^ were assessed using FerroOrange and Mito-FerroGreen, respectively, according to the manufacturers’ instructions. Fluorescence images were acquired by microscopy and relative signal intensity was quantified by image analysis.

### 2.12 Lipid peroxidation assay

Lipid peroxidation was assessed with C11-BODIPY after the indicated transfections or treatments. Oxidized fluorescence was quantified by flow cytometry and/or fluorescence microscopy and compared across groups.

### 2.13 Measurement of GSH, GSSG, the GSH/GSSG ratio, and MDA

GSH, GSSG, the GSH/GSSG ratio, and MDA were measured using commercial kits according to the manufacturers’ protocols and normalized to cell number or protein concentration as appropriate. Kit details are provided in **Supplementary Table S4**.

### 2.14 Immunoprecipitation coupled with mass spectrometry

RAD51-associated proteins were isolated from HEC-1A cells by immunoprecipitation with anti-RAD51 antibody and protein A/G beads, followed by LC–MS/MS analysis.

MS data were searched against the human UniProt database. Protein confidence, sequence coverage, matched/unique peptides, spectral counts, and peak area were used for identification; deubiquitinase candidates were annotated using UniProt and Gene Ontology. USP26 was prioritized based on strong peptide-level evidence (32.2% coverage, 30 unique peptides, 43 spectral counts, peak area 1.07 × 10^7). Full results are provided in **Supplementary Table S5**.

### 2.15 Co-immunoprecipitation

Cells were lysed in immunoprecipitation-compatible buffer and incubated with antibodies against RAD51, USP26, epitope tags, or control IgG; immune complexes were captured with protein A/G beads and analyzed by western blotting. For exogenous assays, tagged RAD51 and USP26 constructs were co-transfected into HEK-293T cells and reciprocal immunoprecipitation was performed with tag-specific antibodies.

### 2.16 Immunofluorescence staining

Cells on coverslips were fixed, permeabilized, blocked, and incubated with anti-RAD51 and anti-USP26 antibodies, followed by fluorophore-conjugated secondary antibodies and DAPI. Fluorescence/confocal microscopy was used to assess subcellular localization and signal overlap.

### 2.17 Cycloheximide chase assay

Cycloheximide was used to block new protein synthesis. Lysates collected at the indicated time points were analyzed by western blotting, and RAD51 abundance was normalized to β-actin and expressed relative to baseline to compare degradation kinetics under different USP26 conditions.

### 2.18 Proteasome inhibition assay

To assess proteasome-dependent RAD51 degradation, cells with USP26 knockdown or overexpression were treated with MG132 or vehicle before protein extraction. RAD51 abundance was then measured by western blotting.

### 2.19 Ubiquitination assay

For ubiquitination assays, cells were transfected with the indicated RAD51, USP26-WT, USP26-C304S, and ubiquitin-related constructs. MG132 was added before harvest to accumulate ubiquitinated proteins. RAD51 was immunoprecipitated and ubiquitination was assessed by anti-ubiquitin or anti-HA western blotting; input lysates were analyzed in parallel.

### 2.20 Xenograft tumor model

Female BALB/c nude mice, aged 6 to 8 weeks, were procured from Henan Skbex Biotechnology Co., Ltd. and housed in a specific pathogen-free (SPF) environment. All animal protocols received approval from the Ethics Committee of Henan Provincial People’s Hospital and were conducted in strict adherence to institutional guidelines.

HEC-1A cells stably transfected with sh-NC, sh-RAD51#1, or sh-RAD51#2 constructs were collected, rinsed, and resuspended in phosphate-buffered saline (PBS) devoid of Matrigel. Each subject received a subcutaneous injection of 6 × 10^6 cells suspended in 200 μL into the right axillary region. Animals were subsequently randomized into experimental cohorts, with five mice assigned to each group.

Tumor dimensions, specifically length and width, were recorded at three-day intervals. Tumor volume was derived using the formula: Volume (mm³) = (length × width²)/2. The study was terminated on day 26, or prematurely if any tumor attained the humane endpoint limit of 1, 500 mm³. Following euthanasia, tumors were surgically resected, documented via photography, weighed, and fixed in 4% paraformaldehyde to facilitate subsequent histological and immunohistochemical (IHC) examinations.

### 2.21 Statistical analysis

All statistical computations were executed via GraphPad Prism (v8.0). Quantitative results are expressed as mean ± standard deviation (SD) unless specified differently. In cellular assays, ‘n’ signifies the count of distinct biological replicates conducted on separate days, excluding technical duplicates such as wells, inserts, or visual fields. Technical repeats were averaged per biological experiment prior to analysis and never counted as independent data points. For animal studies and clinical evaluations, ‘n’ represents individual subjects or specimens, respectively. Each cell-based protocol underwent a minimum of three independent repetitions.

Data normality and variance homogeneity were evaluated via the Shapiro–Wilk and Brown–Forsythe tests, respectively. Two-group comparisons employed an unpaired, two-tailed Student’s t-test for parametric distributions or the Mann–Whitney U test for non-parametric sets. Multi-group analyses utilized one-way ANOVA coupled with Tukey’s *post-hoc* test for all pairwise comparisons or Dunnett’s test when comparing against a single control. Non-parametric multi-group datasets were examined using the Kruskal–Wallis test followed by Dunn’s correction.

Tumor progression trajectories were assessed through two-way repeated-measures ANOVA, incorporating treatment and time as variables, subsequently applying Šídák’s multiple-comparison procedure. Each cohort comprised five animals (n=5). Linear associations for continuous, normally distributed variables were determined using Pearson’s correlation, while Spearman’s rank correlation addressed ordinal or non-normal data. Every statistical evaluation was two-tailed, with significance defined as P < 0.05.

## 3 Results

### 3.1 RAD51 is upregulated in endometrial cancer tissues and cell lines

RAD51 mRNA was higher in UCEC than non-tumor tissues in GSE17025 and GSE146889 (**Figure 1A**), and RAD51 IHC scores were higher in UCEC than adjacent tissues (**Figure 1B**). RAD51 mRNA and protein were also elevated in endometrial cancer cell lines versus hESC cells, with relatively high levels in KLE and HEC-1A (**Figures 1C, D**); these two lines were therefore used for loss-of-function studies.

**Figure 1:**
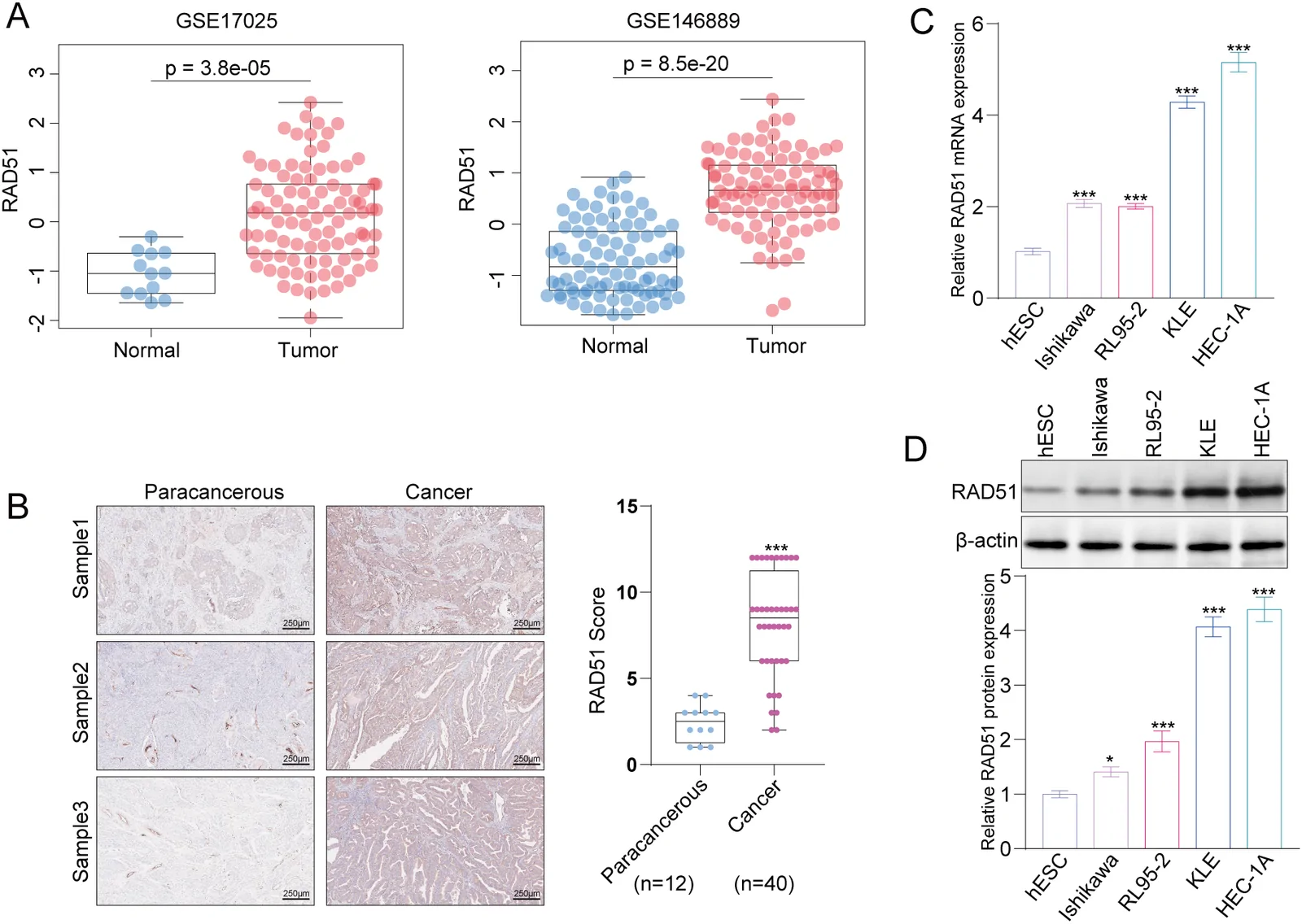
RAD51 is upregulated in endometrial cancer tissues and cell lines. **(A)** RAD51 expression in normal and tumor tissues from GSE17025 and GSE146889. **(B)** Representative RAD51 IHC and score quantification in adjacent non-tumor and UCEC tissues (n = 12 and 40, respectively). Scale bars, 250 μm. **(C)** RAD51 mRNA expression in hESC and endometrial cancer cell lines. **(D)** RAD51 protein expression and quantification. Cell-based experiments were independently repeated three times. Data are mean ± SD. ns, not significant; *P < 0.05; **P < 0.01; ***P < 0.001.

### 3.2 RAD51 knockdown suppresses proliferation and reduces Transwell migration/invasion-associated readouts in endometrial cancer cells

Two independent siRNAs efficiently reduced RAD51 in KLE and HEC-1A cells (**Figures 2A, B**). RAD51 depletion reduced CCK-8 viability and EdU incorporation and increased TUNEL-positive cells (**Figures 2C–E**). Fewer cells reached the lower surface in Transwell assays (**Figure 2F**). Because RAD51 depletion also affected proliferation and cell death, the Transwell data are interpreted as migration/invasion-associated readouts rather than evidence of an independent motility defect.

**Figure 2:**
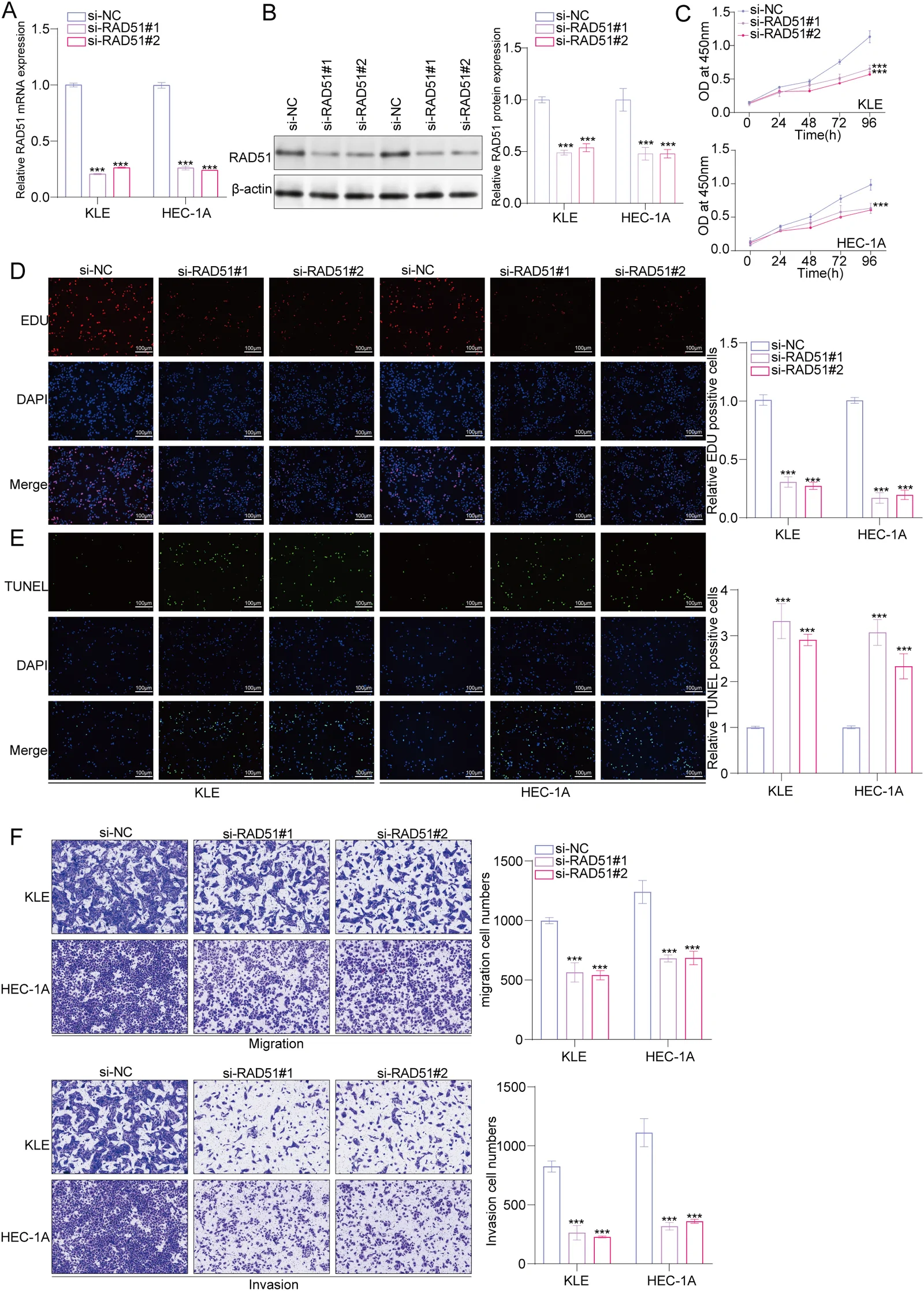
RAD51 knockdown inhibits proliferation, reduces Transwell migration/invasion readouts, and promotes cell death in endometrial cancer cells. **(A, B)** qRT-PCR and western blotting confirming RAD51 knockdown in KLE and HEC-1A cells. **(C)** CCK-8 viability curves. **(D)** EdU incorporation. **(E)** TUNEL staining. **(F)** Transwell migration/invasion assays. Because RAD51 depletion also affects proliferation and cell death, panel F is interpreted as a migration/invasion-associated readout rather than an isolated measure of intrinsic motility. Data are mean ± SD from three independent biological experiments (n = 3). **(C)** was analyzed by two-way repeated-measures ANOVA with Dunnett’s multiple-comparisons test; other quantitative data used one-way ANOVA with Dunnett’s test versus si-NC. ns, not significant; *P < 0.05; **P < 0.01; ***P < 0.001; ****P < 0.0001.

### 3.3 RAD51 is associated with DNA repair and metabolic programs and regulates ferroptosis-related molecules

GSVA linked high RAD51 expression to glutathione metabolism, oxidative phosphorylation, and amino-acid metabolic pathways (**Figure 3A**). Hallmark GSEA across four GEO cohorts consistently associated RAD51 with E2F targets, G2M checkpoint, DNA repair, oxidative phosphorylation, fatty acid metabolism, and reactive oxygen species programs, whereas p53 enrichment varied among cohorts (**Supplementary Figure 1A**). In TCGA-UCEC, RAD51 correlated with representative ferroptosis-related genes (**Figure 3B**). RAD51 knockdown increased TFR1 and ACSL4 and decreased SLC7A11 and GPX4 at both mRNA and protein levels (**Figures 3C, D**), consistent with a ferroptosis-prone state.

**Figure 3:**
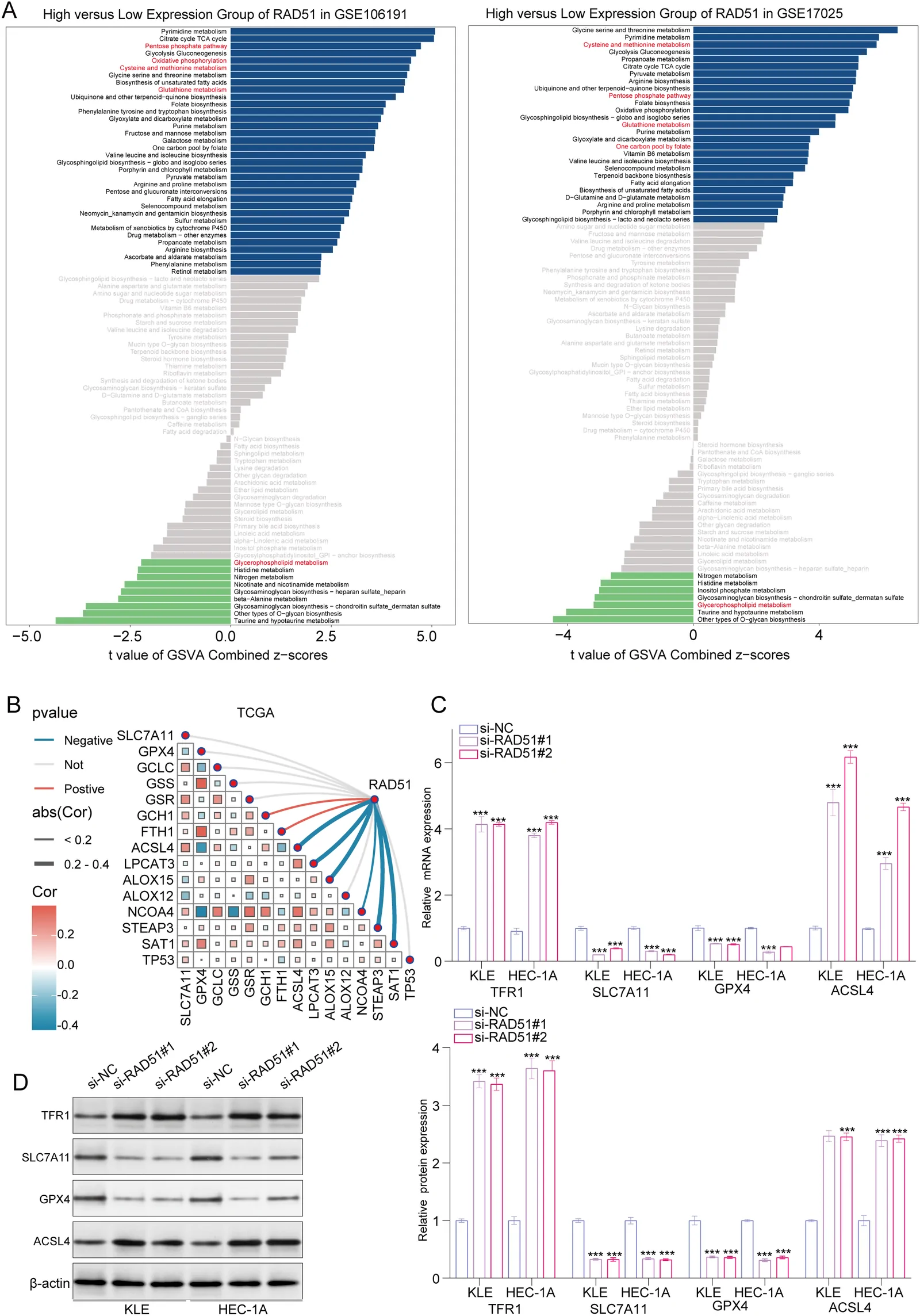
RAD51 regulates ferroptosis-associated metabolic pathways and marker expression. **(A)** GSVA comparing RAD51-high and RAD51-low groups in GSE106191 and GSE17025. **(B)** Correlations between RAD51 and representative ferroptosis-related genes in TCGA-UCEC, assessed by Pearson correlation. **(C, D)** qRT-PCR and western blotting showing increased TFR1/ACSL4 and decreased SLC7A11/GPX4 after RAD51 knockdown. **(C, D)** represent three independent biological experiments (n = 3) and were analyzed by one-way ANOVA with Dunnett’s test versus si-NC. Data are mean ± SD. ns, not significant; *P < 0.05; **P < 0.01; ***P < 0.001; ****P < 0.0001.

### 3.4 RAD51 knockdown promotes iron accumulation, lipid peroxidation, and redox imbalance

RAD51 knockdown increased intracellular and mitochondrial Fe^2+^ (FerroOrange and Mito-FerroGreen; **Figures 4A, B**), enhanced C11-BODIPY oxidation, decreased GSH, increased GSSG and MDA, and lowered the GSH/GSSG ratio (**Figures 4C, D**). RAD51 depletion also increased p21 and phosphorylated p53 without substantially changing total p53 (**Supplementary Figure 1B**). Co-silencing TP53 partially reversed the RAD51-depletion-induced changes in TFR1, ACSL4, SLC7A11, and GPX4 (**Supplementary Figure 1C**), supporting partial involvement of p53-associated stress signaling.

**Figure 4:**
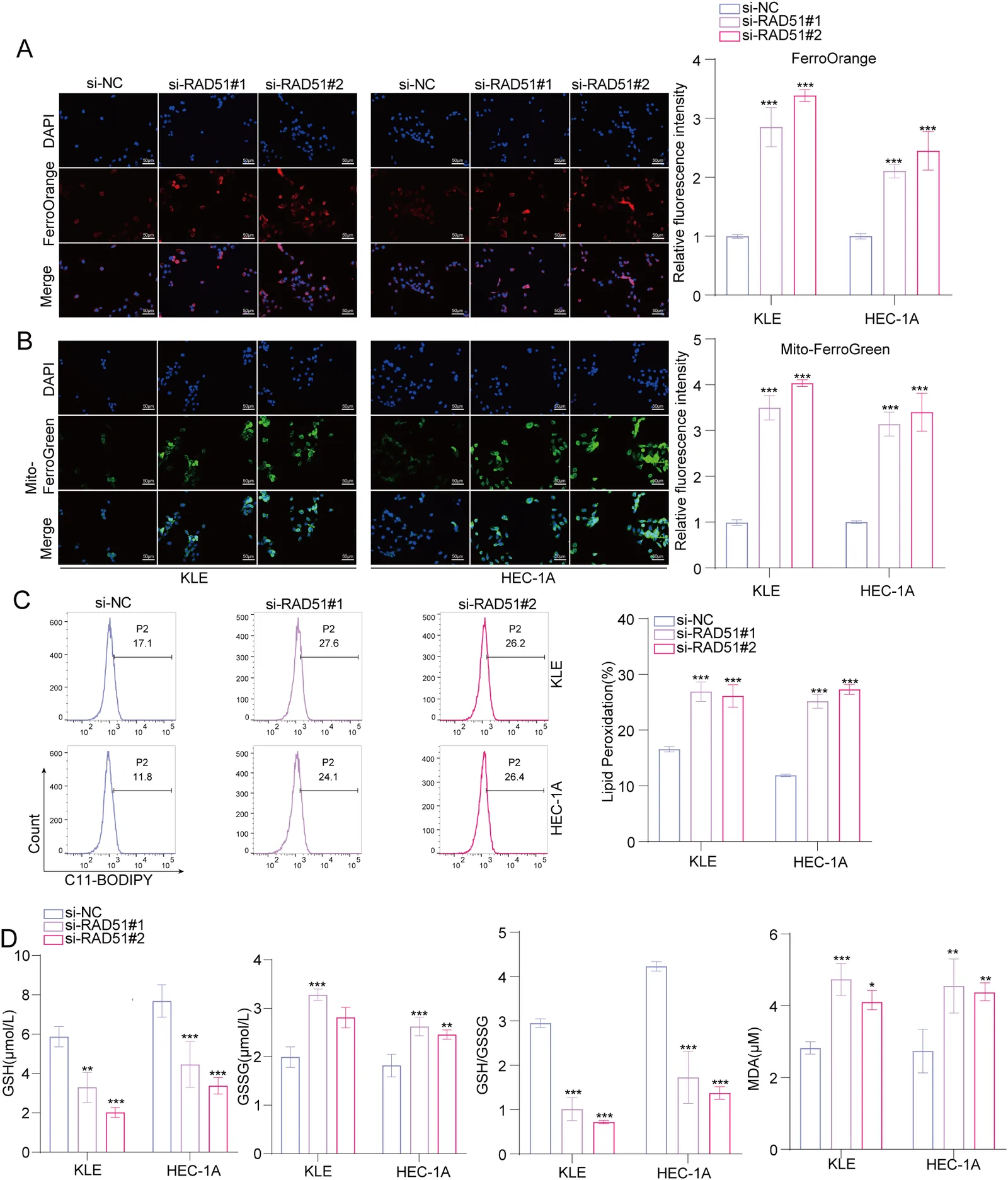
RAD51 knockdown enhances iron accumulation, lipid peroxidation, and redox imbalance. **(A)** FerroOrange staining of intracellular Fe^2+^. **(B)** Mito-FerroGreen staining of mitochondrial Fe^2+^. Scale bars, 50 μm. **(C)** C11-BODIPY lipid-peroxidation analysis. **(D)** GSH, GSSG, GSH/GSSG ratio, and MDA measurements after RAD51 knockdown. Data are mean ± SD from three independent biological experiments (n = 3) and were analyzed by one-way ANOVA with Dunnett’s test versus si-NC. ns, not significant; *P < 0.05; **P < 0.01; ***P < 0.001; ****P < 0.0001.

### 3.5 RAD51 knockdown suppresses endometrial cancer growth and enhances ferroptosis-associated changes *in vivo*

In xenografts, stable RAD51 knockdown slowed tumor growth and reduced final tumor volume and weight (**Figure 5A**). Ki-67 and RAD51 staining decreased, whereas GPX4 decreased and ACSL4 and 4-HNE increased (**Figure 5B**), consistent with enhanced ferroptosis-associated changes. These markers were assessed separately; the results do not imply RAD51–GPX4 colocalization or direct interaction.

**Figure 5:**
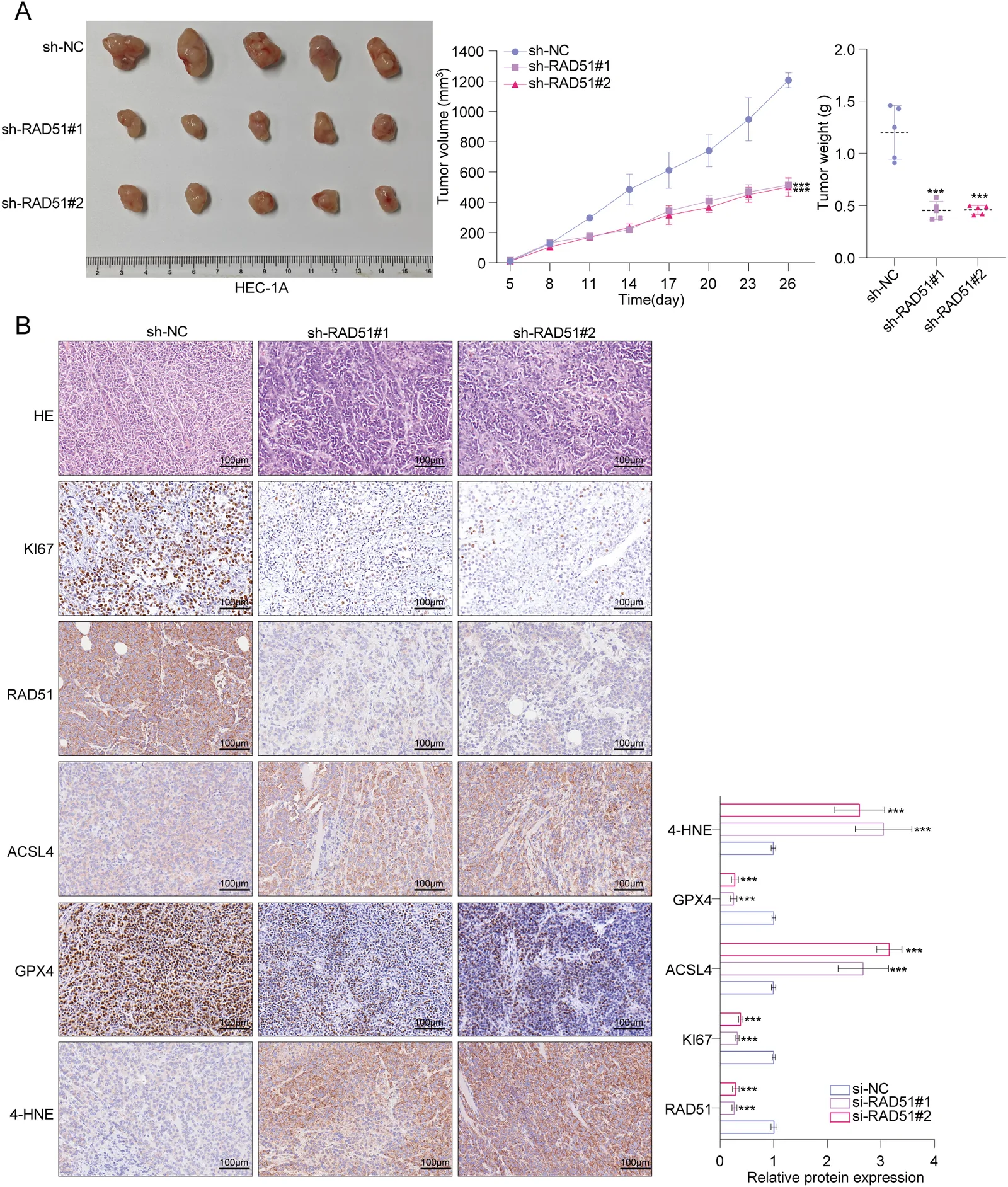
RAD51 knockdown suppresses endometrial cancer growth and promotes ferroptosis-related changes *in vivo*. **(A)** Xenograft images, growth curves, and final tumor weights for HEC-1A cells expressing sh-NC or sh-RAD51. **(B)** HE and IHC staining for Ki-67, RAD51, ACSL4, GPX4, and 4-HNE. The IHC markers were evaluated independently and were not used to assess RAD51–GPX4 colocalization or direct interaction. Scale bars, 100 μm. Five mice were included per group (n = 5). Data are mean ± SD. Growth curves were analyzed by two-way repeated-measures ANOVA with Šídák’s test; final weights and IHC quantification used one-way ANOVA with Dunnett’s test versus sh-NC. ns, not significant; *P < 0.05; **P < 0.01; ***P < 0.001; ****P < 0.0001.

### 3.6 IP–MS identifies USP26 as a candidate regulator associated with RAD51

RAD51 IP–MS identified several deubiquitinase-related proteins. USP26 showed the strongest peptide-level evidence among canonical candidates (−10LgP 42.60; 32.2% coverage; 30 unique peptides; 43 spectral counts; peak area 1.07 × 10^7) and was selected for validation (**Supplementary Table S5**).

USP26 and RAD51 showed partially overlapping localization in HEC-1A cells and were recovered together by endogenous co-IP; reciprocal co-IP of tagged proteins in HEK-293T cells further supported their association (**Figures 6A–D**). Endogenous reciprocal co-IP and partial colocalization were reproduced in KLE cells (**Supplementary Figures 2A, B**). The imaging supports spatial proximity but does not define a compartment-specific mechanism.

**Figure 6:**
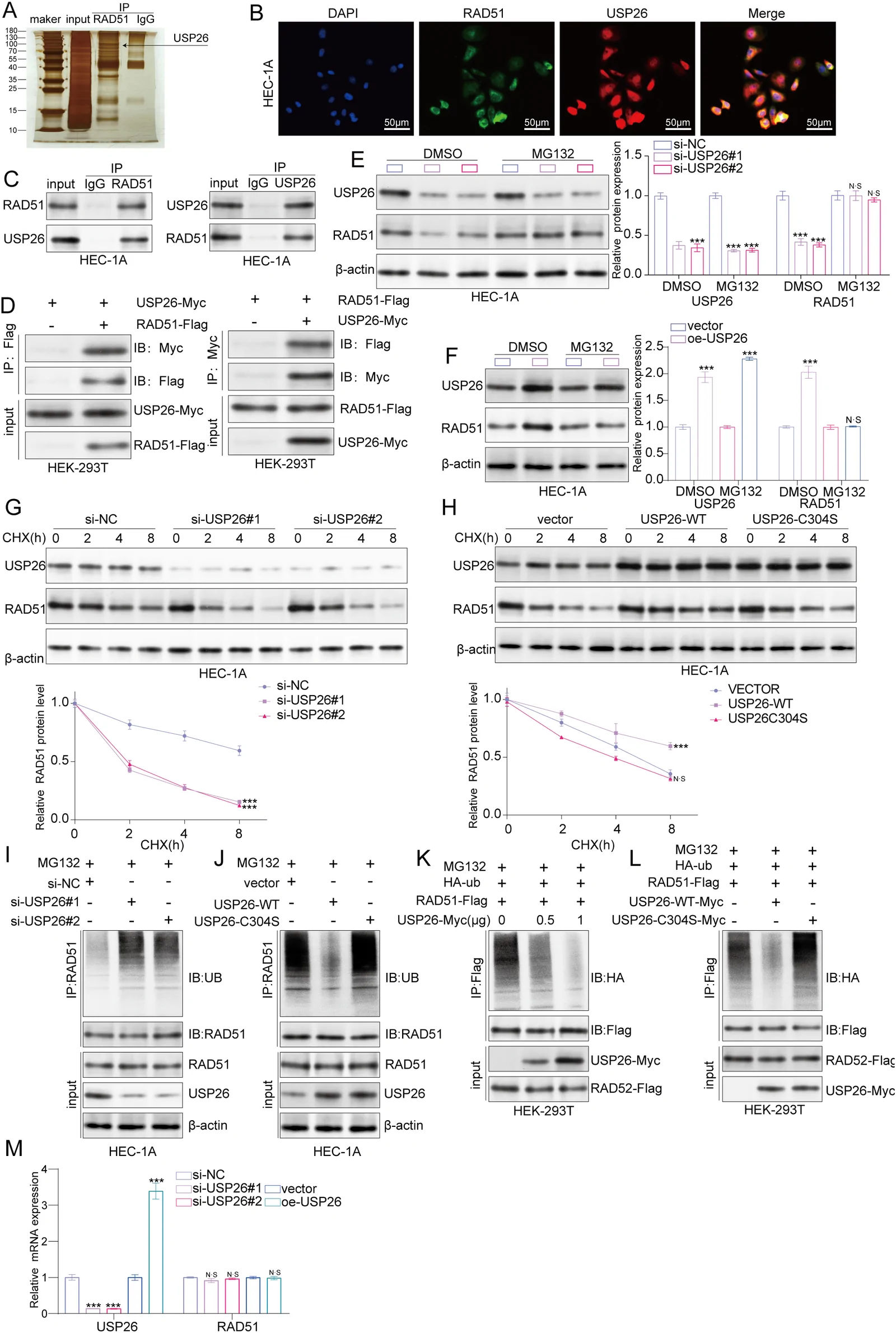
USP26-mediated stabilization of RAD51 promotes endometrial cancer progression and limits ferroptosis-associated stres. **(A)** A Silver-stained RAD51 immunoprecipitates analyzed by LC–MS/MS; full results are in **Supplementary Table S5**. **(B)** Partial USP26/RAD51 signal overlap in HEC-1A cells; this supports spatial proximity but not a compartment-specific mechanism. Scale bars, 50 μm. **(C)** Endogenous co-IP in HEC-1A cells. **(D)** Reciprocal co-IP of tagged USP26/RAD51 in HEK-293T cells. **(E, F)** MG132 experiments after USP26 knockdown or overexpression. **(G, H)** CHX chase after USP26 knockdown or expression of USP26-WT/USP26-C304S. **(I–L)** RAD51 ubiquitination assays. **(M)** qRT-PCR showing that USP26 manipulation does not substantially affect RAD51 mRNA. Quantitative data are mean ± SD from three independent biological experiments (n = 3); panels **(A–D)** and **(I–L)** are representative of three independent experiments. **(E, F)** used two-way ANOVA with Tukey’s test; **(G, H)** used two-way repeated-measures ANOVA with Šídák’s test; **(M)** used one-way ANOVA with Dunnett’s test. ns, not significant; *P < 0.05; **P < 0.01; ***P < 0.001.

### 3.7 USP26 stabilizes RAD51 by preventing proteasome-dependent degradation

USP26 knockdown reduced RAD51 protein, and MG132 restored it; USP26 overexpression increased RAD51, with the difference diminished by MG132 (**Figures 6E, F**). CHX chase showed faster RAD51 decay after USP26 knockdown and prolonged stability with USP26-WT, whereas USP26-C304S was less effective (**Figures 6G, H**). USP26 manipulation did not substantially alter RAD51 mRNA (**Figure 6M**), supporting post-translational regulation.

### 3.8 USP26 reduces RAD51 ubiquitination in cells in a catalytic activity-dependent manner

USP26 knockdown increased RAD51 ubiquitination in MG132-treated cells, whereas USP26-WT reduced ubiquitination and USP26-C304S did not (**Figures 6I, J**). Tagged-ubiquitin assays further showed a dose-dependent reduction in RAD51 ubiquitination by USP26 (**Figures 6K, L**). Together with the stability data, these results support catalytic activity-dependent stabilization of RAD51 through reduced ubiquitination.

### 3.9 RAD51 restoration partially rescues USP26-knockdown phenotypes

In HEC-1A cells, RAD51 re-expression restored RAD51 protein in USP26-depleted cells and partially rescued EdU incorporation and TUNEL phenotypes (**Figures 7A, B**).

**Figure 7:**
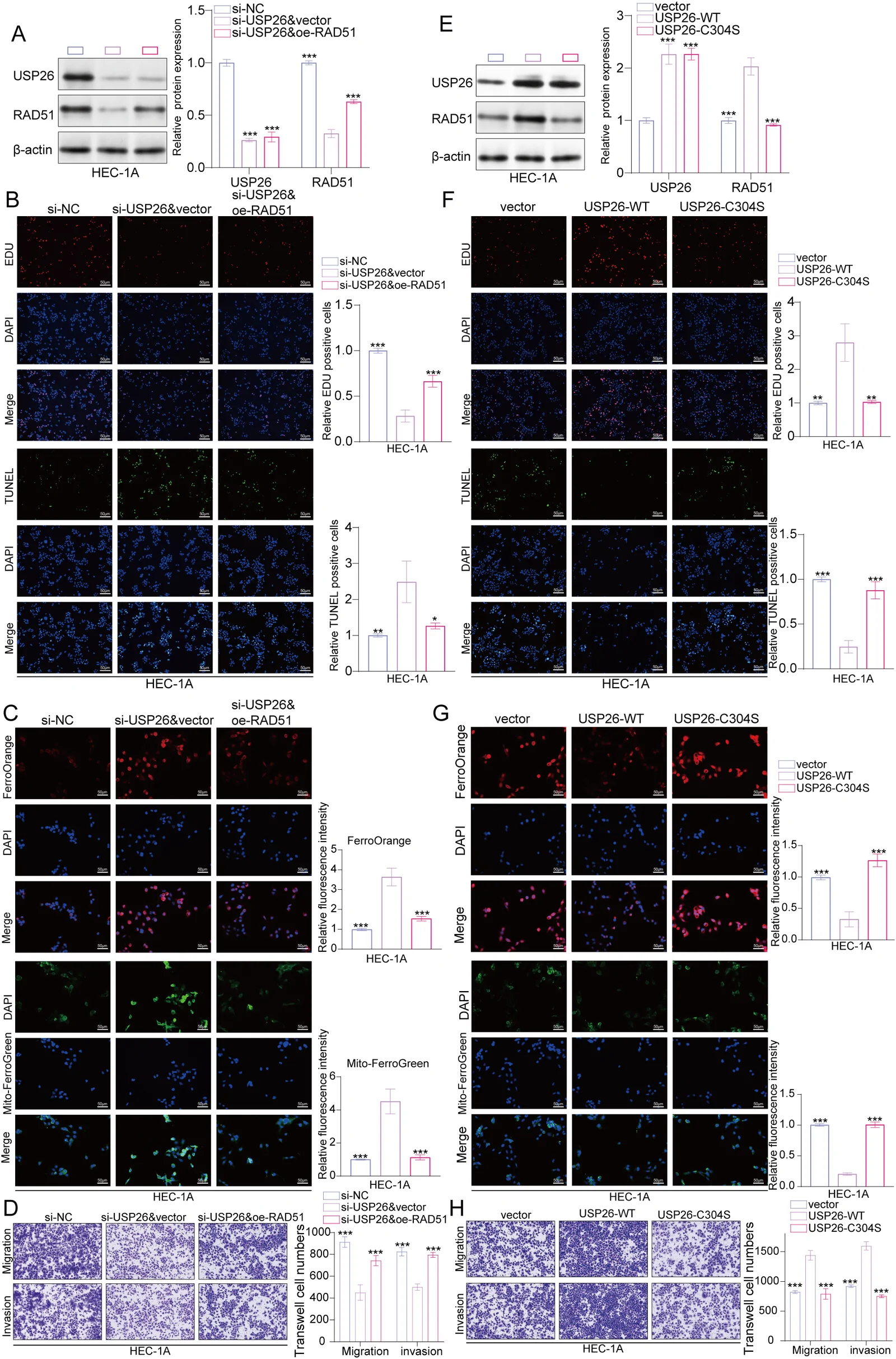
RAD51 mediates USP26-dependent growth and ferroptosis-associated phenotypes and contributes to Transwell migration/invasion readouts. **(A)** RAD51 restoration in USP26-depleted HEC-1A cells. **(B)** EdU and TUNEL analyses. **(C)** FerroOrange and Mito-FerroGreen analyses. **(D)** Transwell assays showing partial restoration by RAD51 re-expression. **(E)** RAD51 stabilization by USP26-WT versus USP26-C304S. **(F)** EdU and TUNEL analyses. **(G)** FerroOrange and Mito-FerroGreen analyses. **(H)** Transwell assays with USP26-WT or USP26-C304S. Because proliferation and cell death are altered in these experiments, panels D and H are interpreted as migration/invasion-associated readouts rather than isolated measures of intrinsic motility. Scale bars, 50 μm. Data are mean ± SD from three independent biological experiments (n = 3). Rescue experiments **(A–D)** were compared with si-USP26 + vector; catalytic-mutant experiments **(E–H)** were compared with USP26-WT. ns, not significant; *P < 0.05; **P < 0.01; ***P < 0.001; ****P < 0.0001.

RAD51 re-expression also attenuated the increases in intracellular and mitochondrial Fe^2+^ and partially restored Transwell migration/invasion readouts after USP26 depletion (**Figures 7C, D**). Because proliferation and cell death were altered concurrently, the Transwell data are not interpreted as an isolated motility effect.

RAD51 restoration partially reversed USP26-depletion-induced lipid peroxidation, GSH/GSSG imbalance, and MDA elevation (**Supplementary Figures 3A, B**), supporting RAD51 as an important downstream mediator of USP26-dependent growth and ferroptosis-associated phenotypes.

### 3.10 USP26 catalytic activity is required for RAD51 stabilization and associated phenotypes

USP26-WT, but not USP26-C304S, markedly increased RAD51 protein (**Figure 7E**), indicating that catalytic activity is required for efficient RAD51 stabilization.

USP26-WT increased EdU incorporation, reduced TUNEL positivity, and decreased intracellular and mitochondrial Fe^2+^; these effects were attenuated with USP26-C304S (**Figures 7F, G**).

USP26-WT also increased Transwell migration/invasion readouts and reduced lipid peroxidation, GSSG, and MDA while increasing GSH and the GSH/GSSG ratio; USP26-C304S showed weaker effects (**Figure 7H**; **Supplementary Figures 3C, D**). The Transwell findings are interpreted in the context of concomitant proliferation and cell-death changes. These data support a requirement for USP26 catalytic activity in RAD51 stabilization and the associated phenotypes.

### 3.11 USP26 and RAD51 protein expression in UCEC tissues

USP26 staining tended to be higher in RAD51-high tumors (**Figure 8A**), whereas USP26 and RAD51 mRNA levels were not significantly correlated in GSE106191 or GSE17025 (**Figure 8B**). In 40 UCEC specimens, USP26 and RAD51 IHC scores were analyzed by Spearman rank correlation (Spearman ρ = 0.3939, P = 0.0270; **Figure 8C**). This tissue-level result is interpreted as supportive rather than causal and is compatible with the post-translational mechanism indicated by the biochemical data.

**Figure 8:**
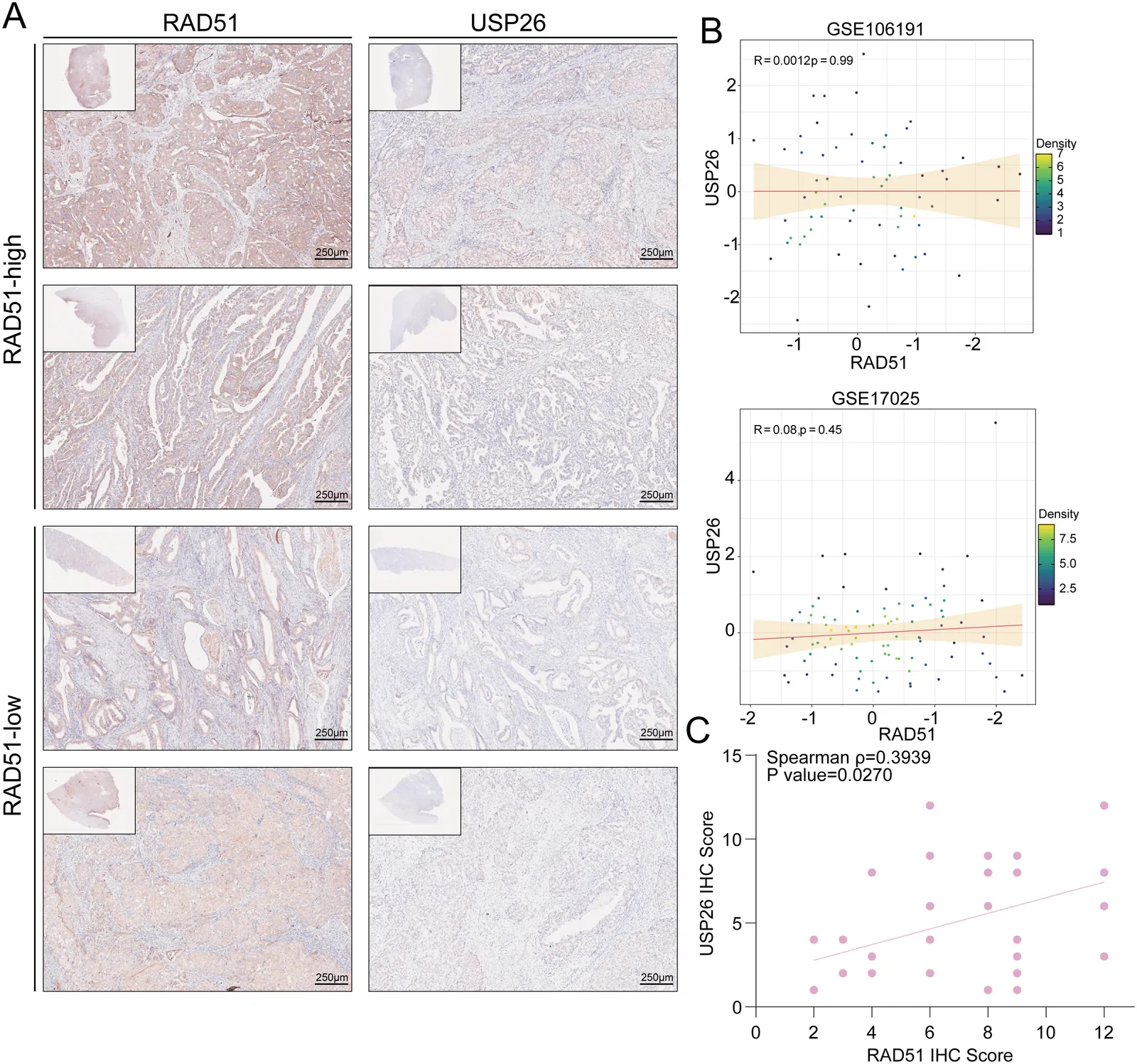
USP26 and RAD51 protein expression in endometrial cancer tissues. **(A)** Representative USP26 and RAD51 IHC in RAD51-high and RAD51-low UCEC tissues. Scale bars, 250 μm. **(B)** USP26–RAD51 mRNA correlations in GSE106191 and GSE17025. **(C)** Spearman rank correlation of USP26 and RAD51 IHC scores in 40 UCEC tissues (ρ = 0.3939, P = 0.0270). The tissue-level result is interpreted as supportive rather than causal.

## 4 Discussion

We identify USP26 as a post-translational regulator of RAD51 in endometrial cancer. RAD51 depletion suppressed proliferation and xenograft growth and was accompanied by lower Transwell migration/invasion readouts, increased iron accumulation and lipid peroxidation, and impaired glutathione redox balance. USP26 associated with RAD51, reduced its ubiquitination, and stabilized it in a catalytic activity-dependent manner; RAD51 re-expression partially rescued the effects of USP26 depletion. Together, these findings support a USP26–RAD51 axis linking RAD51 protein stability to ferroptosis-associated stress.

RAD51 is best known for homologous recombination and tumor-cell tolerance of replication stress (30). Here, RAD51 depletion increased p21 and phosphorylated p53 and altered multiple ferroptosis-related proteins; TP53 co-silencing partially reversed these changes. Combined with pathway associations involving DNA repair, oxidative stress, metabolism, and p53 signaling, the data support partial involvement of p53-associated stress signaling. However, they do not establish a complete DDR-to-ferroptosis pathway or direct transcriptional control of SLC7A11, GPX4, TFR1, or ACSL4 by RAD51.

RAD51 depletion also increased cellular and mitochondrial Fe^2+^, lipid peroxidation, MDA, and 4-HNE while reducing GSH-associated antioxidant capacity and GPX4/SLC7A11 expression. Similar changes occurred in xenografts, indicating a broader shift toward ferroptosis susceptibility rather than alteration of a single marker. RAD51 and GPX4 were assessed as separate IHC markers; their different subcellular distributions therefore do not imply or require colocalization.

Mechanistically, USP26 depletion reduced RAD51 protein without a comparable mRNA change, MG132 restored RAD51, and CHX chase showed altered RAD51 turnover. USP26 also reduced RAD51 ubiquitination, whereas USP26-C304S was less effective at both deubiquitination and stabilization. These findings support a catalytic activity-dependent ubiquitin–proteasome mechanism and are consistent with known ubiquitin-dependent regulation of RAD51 (21–23, 31, 32). The CHX assays were designed to measure RAD51 turnover and do not imply reciprocal regulation of USP26 stability (33).

USP26 and RAD51 showed partial nuclear overlap, compatible with the nuclear role of RAD51, but immunofluorescence cannot define where deubiquitination occurs. Thus, colocalization is interpreted as evidence of spatial proximity only; compartment-specific studies will be needed to resolve this issue.

The catalytic-mutant and rescue experiments suggest that RAD51 is an important, but not necessarily exclusive, mediator of USP26 function. Pharmacological inhibition of USP26 could therefore destabilize RAD51 and increase ferroptosis susceptibility, but this possibility requires selective inhibitors and formal pharmacological validation.

Clinical and public-dataset analyses provide supportive rather than causal evidence. RAD51 was elevated in UCEC, and multi-cohort GSEA associated RAD51 with cell-cycle, DNA-repair, oxidative-stress, and metabolic programs. USP26–RAD51 mRNA correlations were not significant in the analyzed GEO datasets, whereas their protein-level relationship was evaluated by Spearman correlation in the clinical cohort. This pattern is compatible with post-translational regulation but requires independent clinical validation.

Therapeutic implications should be considered cautiously because RAD51 is required for genome maintenance in normal proliferating cells. Whether targeting USP26 can preferentially reduce RAD51 in tumor cells is unknown; tumor selectivity, therapeutic window, normal-tissue safety, and potential combinations with ferroptosis-inducing agents require preclinical evaluation.

Several limitations remain. The clinical cohort was single-center and requires independent multicenter validation. The RAD51 ubiquitin-chain linkage(s) and lysine site(s) targeted by USP26 were not mapped. The mechanism connecting RAD51 loss to p53-associated stress and ferroptosis-related changes remains incomplete, and the subcellular site of USP26-mediated RAD51 regulation is unresolved. In addition, Transwell assays are confounded by concurrent changes in proliferation and cell death and therefore do not isolate intrinsic motility. Future work should address these mechanistic questions and evaluate selective USP26 inhibition in patient-derived models.

Overall, USP26 stabilizes RAD51 by reducing its ubiquitination, thereby supporting endometrial cancer growth and resistance to ferroptosis-associated stress. These findings provide a mechanistic basis for further investigation of the USP26–RAD51 axis, while its therapeutic selectivity and safety remain to be established.

## Data Availability

The original contributions presented in the study are included in the article/**Supplementary Material**. Further inquiries can be directed to the corresponding author.

## Supplementary material

Supplementary Figure 1 RAD51 depletion activates p53-associated stress signaling and alters ferroptosis-related proteins. (A) Hallmark GSEA heatmap across GSE106191, GSE119041, GSE146889, and GSE17025. Positive and negative normalized enrichment scores indicate positive and negative association with RAD51, respectively; asterisks indicate GSEA significance levels. (B) Western blotting and quantification of RAD51, p21, total p53, and phosphorylated p53 after RAD51 knockdown in KLE and HEC-1A cells. (C) Western blotting and quantification of TFR1, SLC7A11, GPX4, and ACSL4 in cells transfected with si-NC, si-RAD51#1, or si-RAD51#1 + si-TP53. β-actin was the loading control. Panels B and C show three independent biological experiments (n = 3); panel B was analyzed by one-way ANOVA with Dunnett’s test and panel C by one-way ANOVA with Tukey’s test. For panel A, significance was based on the GSEA output. ns, not significant; *P < 0.05; **P < 0.01; ***P < 0.001; ****P < 0.0001.

Supplementary Figure 2 Endogenous association and colocalization of USP26 and RAD51 in KLE cells. (A) Reciprocal endogenous co-immunoprecipitation in KLE cells with species-matched IgG controls. (B) Representative RAD51 (green), USP26 (red), DAPI (blue), and merged immunofluorescence images. Scale bars, 50 μm. Images are representative of three independent experiments (n = 3); no statistical analysis was performed for these qualitative experiments.

Supplementary Figure 3 RAD51 restoration and USP26 catalytic activity regulate lipid peroxidation and redox balance. (A) C11-BODIPY lipid-peroxidation analysis after USP26 knockdown with vector or RAD51 rescue. (B) GSH, GSSG, GSH/GSSG ratio, and MDA in the rescue groups. (C) C11-BODIPY analysis in vector, USP26-WT, or USP26-C304S cells. (D) GSH, GSSG, GSH/GSSG ratio, and MDA in these groups. Data are mean ± SD from three independent biological experiments (n = 3) and were analyzed by one-way ANOVA with Tukey’s multiple-comparisons test. ns, not significant; *P < 0.05; **P < 0.01; ***P < 0.001; ****P < 0.0001.
